# Keyword index to Volume 82

**DOI:** 10.1054/bjoc.2000.1285

**Published:** 2000-05-18


					Aberrant crypt foci 1276
ACE 550
Acetaminophen 1364
Activin 112, 1415
Activin receptors 112, 1415
Activity 381
Acute lymphoblastic leukaemia 1568
Acute myeloid leukaemias 1387
ADCC 441
Additive effects 46
Adenocarcinoma 412, 865, 1510
Adenoma-carcinoma sequences 9, 1276
Adenovirus vectors 642
Adhesion 472
Adhesion proteins 195
Adjuvant chemotherapy 1920
Admixtures 1510
Adolescents 1636
Adrenal androgens 1577
Advanced colorectal cancer 1789
Advanced pancreatic cancer 1772
Advanced stage III 1396
Advanced stage IV 1396
Adverse effects 777
Aetiocholanolone 1577
Aetiology 1073, 1117
Africa 1585
AgNOR 1724
AIDS 1585
Alcohol 1892
Alcohol consumption 204
Alkaline phosphatase 881
Allelic loss 543
Alpha-particles 763
Amino acid exchange rates 399
Aminoanthraquinones 1300
Anaemia 93
Analgesics 1364
Anaplastic astrocytomas 1371
Androgen receptors 39
Androstenedione 1867
Androsterone 1577
Angiogenesis 161, 591, 844, 1004, 1427,
1441, 1528, 1974
Animals 900
Anthracycline resistance 529
Anti-androgens 283
Antibiotics 1107
Antibodies 85
Anticancer drugs 1851
Antigen presentation 1474
Anti-HER2 antibodies 46
Anti-LeY antibodies 441
Antimyosin scintigraphy 777
Antineoplastics 1789
Antineoplastic agents 93
Anti-oestrogen 46
Antisense EWS-Fli-1 16
Anti-tumour activity 480
Anti-tumour effect 1469
AP-1 1041
AP-2 2017
AP-2 1983
APC 9
APC genes 348
Apoptosis 46, 131, 368, 452, 459, 467, 579,
905, 981, 1145, 1177, 1198, 1441, 1459,
1553, 1702, 1709, 1724, 1747, 1819, 1827,
1851, 1993
AQ4N 1469, 1925
Arachidonic acid 2002
Archival Pap smears 1421
Array hybridization 1149
Aspirin 1364
Assessments 800
Astrocytomas 543
Ataxia-telangiectasia 1945
Athletes, former college 726
ATM 1945
ATP 1776
Avidin-biotin 616
Azaserine 900
Basic fibroblast growth factor 39, 1004
B-cell non-Hodgkin's lymphoma 1396
BCL-2 436, 579, 1441, 1446
bcl-2 expression 270
Bcl-2 protein 418
Bcl- 178
Betel nut chewing 1871
Binding proteins 1561
Biomarkers 1223, 1625, 1627, 1671
Bioreduction 1925
Bioreductive agent potentiation 1776
Bioreductive drugs 651
2,3-Bisphosphoglycerate phosphatase 20
Bisphosphonates 858, 1381, 1459
Bladder cancer 136, 161, 1364, 1553, 1959
Bladder washings 136
Blood flow 2009
Blood transfusion 93
Blood vessel invasion 404
Bombesin 124
Bone markers 858
Bone marrow 953, 1290
Bone metastases 858, 1381
Book reviews 250, 499
Borderline tumours 760
Borocaptate sodium 1764
p-Boronophenylalanine 1764
Brain tumour invasion 52
BRCA1 151, 538, 705
BRCA1 1249, 1266
BRCA2 151, 553, 705
BRCA2 1249, 1266
Breast cancer 20, 46, 151, 171, 354, 361,
368, 381, 452, 514, 518, 529, 538, 553,
683, 726, 742, 777, 844, 1107, 1163, 1204,
1312, 1600, 1625, 1656, 1844, 1867, 1897,
1920, 1965
Breast cancer, human 142, 446, 666
Breast cancer cells 1290, 1459
Breast cancer genetics 705
Breast cancer incidence 1070
Breast cancer management 1619
Breast cancer risk 1577
Breast cysts 492
Breast cyst fluid 492
Breast neoplasms 339, 1914
Breast symptoms 742
Breast-ovarian cancer syndrome 705
Bronchial mucosa 412
Bronchus 418
Butyrate 195
CA 125 1535
CA 19-9 1013
Calcium influx 39
Calman-Hine 208
Camptothecin 1827
Cancer chemotherapy 966
Cancer registration 1111
Cancer registries 712, 1585, 1863
Cancer vaccines 1619
Canine 1297
Carbogen 2009
Carbonic anhydrase 1808
Carcinogenesis 1276, 1795
Carcinomatous effusion 621
Cardiac sarcomas 1183
Carnitine 399
Case-control studies 204, 227, 1860
Caspase 1993
-Catenin 1689
Cathepsin D gene 1844
Cathepsin H 782, 1317
CD3-directed bispecific antibodies 472
CD34 339
CD44 1283
CD95 (APO-1/Fas) 1851
CD95 ligands 1446
CDK 675
cdk4 818, 1271
Cell adhesion 1717
Cell adhesion molecules 1290, 1808
Cell cycles 1123, 1945
Cell cycle arrest 642
Cell growth 1480
Cell lines 1510
Cell proliferation 270, 1123
c-erbB-4 1163
Cervic carcinoma 1177
Cervical cancer 1348, 1709
Cervical intraepithelial neoplasia 1332
Cervix 424
c-fos 2002
CFU-GM growth inhibition 524
CGH 65
Charge 1513
Chemoradiation 98
Keyword index to Volume 82
2032
British Journal of Cancer (2000) 82(12), 2032-2036
(c) 2000 Cancer Research Campaign
doi: 10.1054/ bjoc.2000.1285, available online at http://www.idealibrary.com on
Keyword index 2033
British Journal of Cancer (2000) 82(12), 2032-2036
(c) 2000 Cancer Research Campaign
Chemosensitivity 1186
Chemotherapy 777, 806, 1013, 1107, 1158,
1371, 1897, 1932, 1959
Chernobyl 315
Childhood 1396
Childhood acute lymphoblastic leukaemia
234
Childhood cancer 1073, 1939
Childhood leukaemia 255, 1353, 1568, 1571
Childhood lymphoma 1353
Children 251, 702, 1339, 1636, 1939
Chinese 538
Chromosomal aberrations 1407
Chromosomal in situ hybridization 1407
Chromosomes 424
Chromosome 1 1204
Chromosome 7 323
Chronic myeloid leukaemia cells K562 1480
Cigarettes 1892
Cigarette smoke 782
Circulating tumour cells 118, 157
Cisplatin 98, 104, 300, 488, 1925, 1932
c-jun N-terminal kinase-1 1993
c-Kit 1453
Clinical benefit responses 1772
Clinical definition 529
Clinical prostate cancer 731
Clinical trials 213
Clinical tumour sizes 404
Clinicians' opinions 213
Clonal heterogeneity 543
Clonality 601, 827
Clustering 1571
c-met 945
CMF 1920
Collagenase-3 657
Colon cancer 195, 1276, 1510, 1717, 1724,
1860
Colorectal adenomas 348
Colorectal cancer 9, 124, 178, 560, 871,
1004, 1009, 1283, 1317, 1539, 1689
Colorectal cancer, advanced 1789
Colorectal cancer, human 913
Colorectal neoplasms 535, 905
Combination chemotherapy 1547, 1914
Combined chemoradiotherapy 104
Communication 1783
Comparative genomic hybridization 1218
Completeness 1111
Compound biological effectiveness factor
1764
Cost-effectiveness 81
Covalent binding 1300
CpG island 131
CPT-11 913
Cranial irradiation 255
Creatine kinase 20
Cryosurgery 794
Curability 1186
Cutaneous melanomas 2017
Cyclins 642
Cyclin D 675
Cyclophosphamide 278, 1469, 1925
CYP19 1247
Cysteine proteinases 931, 1317
Cystine knot growth factor 1553
Cytochrome P450 1646
Cytochrome P450 reductase 651
Cytochrome P4501A1 852
Cytogenetic alterations 1239
Cytogenetics 330
Cytokeratin 20 157
Cytokines 1009, 1233
Dartmouth regimen 1759
Data matching 712
Day care 234
DCC 459, 571
Death certificate only 712, 1111
Deletion mapping 1801
Depression 1249
Deprivation 1568
Desmethyltamoxifen 1629
Desmoids 827
Dexamethasone 1709
Diagnosis 1022
Diagnostic accuracy 1017
Diet 204
Diethylnitrosamine 467
Differential diagnosis 1271
Differential gene expression 1149
Differentiation 881, 1327
Dipyridamole 924
Disease models 900
Distribution 348
DNA 142
DNA cross-linking 1300, 1305
DNA damage 1819
DNA damaging agents 28
DNA hypermethylation 514
DNA repair deficiency 629
Dose intensities 1914, 1920
Doxorubicin 973
Drug deliveries 1513
Drug designs 1808
Drug resistance 488, 1732, 1819
Drug sensitivity 34
DTIC 1759
Dyspnoea 800
Early or delayed treatment 1254
Early detection 1605
Early squamous cell carcinoma 418
E-cadherin 195
E-cadherin mutations 568
Economic development 1875
Ecteinascidin-743 1732
eIF-4E 161
Elderly patients 270
ELISA 85, 782
Encephalopathy 291
Endometrial cancer 459, 675
Endometrium 1030
Endothelial cells 657
Enolase 20
Epidemiology 718, 1600
Epidermal growth factor 1041
Epidermal growth factor receptors 186, 1993
Epigenetic radiation mechanisms 1740
Epirubicin 777, 806, 1914, 1932
Epithelia-stroma interactions 990
Epithelial cells 657
Epithelial ovarian cancer 1415, 1535, 1983
Epithelial ovarian neoplasms 1446
Epstein-Barr virus 1117
Erb-3 683
ERBB-2 666, 683
Erythropoietin 93
ESHAP 278
Ethnic groups 1339, 1568
Etoposide 488
Ewing's sarcoma 16
Exon v6 1283
Familial 241, 535, 1795
Familial adenomatous polyposis 348, 827,
1689
FAP 348, 827, 1689
Fas 467, 1211, 1682, 1827
Fas ligands 1211, 1446
FASAY 1145
FasL 1682
Fatigue 789
FcR 441
Fecundity 737
Females 204
FHIT 838, 1191
Fibroblasts 657
FISH 1204
Fit-1 635
5-Fluorouracil 98, 104, 772, 1772
Follistatin 112
Fotemustine 488
Founder BRCA1/2 mutations 705
Founder effects 553, 705
Free hCG 1553
Free prostate-specific antigens 731
Functional adrenocortical tumours 1035
Functional imaging 1223
Gastric adenocarcinoma 1814
Gastric adenoma 1814
Gastrin releasing peptides 124
Gastritis 1814
Gastro-oesophageal cancer 1932
Gastro-oesophageal junctions 865
Gemcitabine 806, 1013, 1772
Gender 1070
Gene alterations 838
Gene expression 2002, 2017
Gene rearrangements 315
Gene silencing 131
Genetic counselling 1249
Genetic polymorphism 852
Genetic susceptibility 1646
Genomic imprinting 753
Genotype analysis 705
Geographic variations 446
Germline 568
Germline mutations 348, 818, 1403
Gestational trophoblastic disease 1547
GH-RH antagonists 1724
Glandular kallikrein, human 361
Glioblastomas 543
Glioblastoma multiforme 1371
Gliomas 74, 480, 608, 635, 1218, 1974
Glioma cells, human 28
-D-Glucosyl-ifosfamide mustard 629
Glutamate transport 399
Glutathione 399
Glutathione S-transferase 1646
Grading 1656
Granulocyte colony-stimulating factor 1485,
1920
Growth 241
Growth factors 52
Growth factor receptors 1163
Growth fraction 579
Growth inhibition 16
Growth substances 93
2034 Keyword index
British Journal of Cancer (2000) 82(12), 2032-2036 (c) 2000 Cancer Research Campaign
GTPase 1035
H19 753
Haematotoxicity 524
Haemophilus influenzae 1261
Head and neck cancer 392, 757
Heat shock protein 90 85
Height 241
Helicobacter pylori 1814
Heparin-binding growth factors 1233
Hepatoblastomas 753, 1561
Hepatocarcinogenesis 467
Hepatocellular carcinoma 833, 1211
Hepatocyte growth factor 891, 945
Heregulin 683
Hevin 1123
HGF 891, 945
High-dose chemotherapy 81, 524, 777
High-grade gliomas 1371
Histamine 167
Histology 227
Histopathology 1204
HLA 1348
HLA class I 1058
hMLH1 871
hMSH2 871
Hodgkin's disease 81, 1117
Home enteral tube feeding 263
Homing 953
Hormone replacement therapy 1860
Hormone resistance 186
Hormone responsiveness 270
Hospitals 208
HPPH 1297
HPV 392, 757
17-HSD 518
hTEP1 833
hTERT 833, 1051, 1819
Human herpesvirus 8, detection of antibodies
to 702
Human papillomavirus 1421, 1709
HUMARA 827
HUVECs 385
Hydralazine 2009
4-Hydroxytamoxifen 1629
Hyperbaric oxygenation 88
Hyperfractionated thoracic radiation 104
Hyperplasia 1953
Hypoxanthine transport 924
Hypoxia 635, 651, 937, 1177, 1528
Idarubicin 524
Idarubicinol 524
Identity-by-descent 553
Ifosfamide 291, 300, 1636
IB- 28
Immune responses 691
Immune selection 1900
Immune suppression 1009
Immunochemotherapy 772
Immunocytochemistry 1965
Immunohistochemistry 374, 518, 1022, 1030,
1191, 1211, 1446, 1528, 1689
Immunotherapy 472, 1058, 1474
In situ hybridization 124
In vitro cell interactions 472
In vivo cell interactions 931
Incidence 446, 737
Incinerators 1103
Index 381
Inductively coupled plasma mass spectometry
966
Infections 234, 1571
Infectious agents 1117
Infecundity 737
Infertility 737
Influenza 1261
Influenza virus 1474
Inhibin 112
Inhibition 1519
Insulin-like growth factors 1561
Insulin-like growth factor-I 241, 1724
Insulin-like growth factor-I receptors 1724
Insulin-like growth factor-II 385, 753, 1724
Integrins 1433
v
3
-Integrin 1974
Intellectual educational sequelae 255
Interactions 1290, 1519
Interferon- 772
Interferon-type II 1138
Interleukin-2 772, 937
Interleukin-4 1717
Interleukin-6 621, 881, 1312
Interleukin-13 1717
Interstitial collagenase 657
Intestinal mucositis 945
Intracerebral therapy 74
Invasion 385, 666, 891, 1063
Ion microscopy imaging 1764
Ionizing radiation 308
Ischaemia-reperfusion injuries 1835
Isoforms 1694
Isolated hepatic perfusion 1539
Isolated limb perfusion 973, 1000
Italian children 702
Japanese-Americans 1867
Kallikrein gene family 361
Keratinocytes 424
Ki-67 167, 621
Kidney 550
Ki-S2 1051
Ki-S5 1051
K-ras 9
K-ras mutants 1035, 1183
KSHV 702
Large-core needle biopsies 1017
Lasers 56
Late host exposure models 1117
Late treatment 1393
LCIS 568
Letters to the Editors 246-249, 497-498,
998, 2024
Leucovorin 98
Leukaemia 1339
Leukaemia, acute lymphoblastic 1568
Leukaemia, acute myeloid 1387
Leukaemia classification 1073
Leukoplakia 1433, 1871
Leydig cells 789
LH39 844
Li-Fraumeni syndrome 1145, 1939
Lichen 1433
Life years 220
Lifestyle 1358
Lipoma 1271
Liposarcoma lipoma-like 1271
Liver cancer 1103
Liver metastases 1539
Liver microcirculation 794
Liver neoplasms 1041
Long-term survival 404
Longitudinal studies 1107
Loss of heterozygosity 323, 1191, 2017
Low penetrance 757
Low rectal cancers 1131
Lung cancer 65, 227, 374, 1191
Lung squamous cell carcinoma 852
LY231514 924
Lymph nodes 295
Lymphadenectomy 295
Lymphoma 81, 278
Lymphoscintigraphy 295
Macromolecules 1513
Magnetic fields 1073
Magnetic resonance imaging 88, 2009
Male breast cancer 1247, 1266
Malignancy 1327
Malignant lymphoma 601
Malignant melanoma 1149
Mammary epithelial cells, human 666
Mammary tumour virus, mouse 446
Mammographic screening 220
Manganese superoxide dismutase 1022
Manumycin 905
MAP kinases 891, 1041
Mast cells 167
Matrix metalloproteinases 52, 960
mdm2 392, 1271
MDR1 171
Mechanism of activation 1300, 1305
Melanocytes 1453
Melanocytic naevi 1149
Melanomas 488, 1051, 1158, 1453, 1593,
1605, 1759, 1887
Melarsoprol 452
Melphalan 1000, 1539
Menopause 1577, 1860
Merkel cell carcinoma 823
Mesenchymal stem cells 1290
Mesothelial cells 1233
Mesothelioma 1022
Meta-analysis 1017, 1789
Metabolic rate of glucose 608
Metabolism 1300, 1519
Metallothionein immunohistochemistry 1198
Metaplasia 1953
Metastasis 195, 1239, 1694
Metastatic adenocarcinoma 1022
Methotrexate 945
Methoxymorpholino doxorubicin 767
Methylation 131
Methylene Blue 291
Microcirculation 900
Microdissections 838
Microevolutionary models 1900
Micrometastases 1283, 1290
Microsatellites 1557
Microsatellite analysis 543
Microsatellite instability 1276, 1814
Microsomal epoxide hydrolase 852
Migration 385
Minimal residual disease 118
Misonidazole 635
Mitochondria 1740
Mitomycin C 1305
Mitoxantrone 1300
Keyword index 2035
British Journal of Cancer (2000) 82(12), 2032-2036
(c) 2000 Cancer Research Campaign
MMTV 446
Mobilization 278
Molar pregnancies 1393
Molecular diagnostics 1650
Molecular oxygen 88
Monoclonal antibodies 616, 1058, 1993
Monocytes 441
Moods 789
Mortality 1887
Motility 891
Mouse 467
MRGlu 608
MRI 88, 2009
mRNA in situ hybridization 2017
MRP 171
MRS 1776
MSI 913
Mucin 691
Multicentre studies 1138
Multicentric renal cell tumours 1407
Multidimensional 800
Multidrug resistance 1327, 1851
Multiple myeloma 953, 1254
Multiplex assay 171
Murine tumours 937
Mus domesticus 446
Mutations 136, 553, 763, 823, 1266, 1276
Mutation analysis 151
Myeloma 1261
Myogenic transcription factors 1239
NAD(P)H 1305
Nasopharyngeal cancer 1198, 1953
Natural compounds 1732
Neoadjuvant therapy 98
Neoplasms 827, 1107, 1900
Neovascularization 339
Nephrotoxicity 1636
Neuroblastoma 1171, 1801
Neuromedin B 124
Neurotoxicity 966
Neutrophils 1485
NF-B 28
Nicotinamide 2009
Nitric oxide 1835
Nitric oxide synthase inhibitors 1835
nm23 1662
Node-negative breast cancer 404
Non-compliance 251
Non-Hodgkin's lymphoma 1344
Non-Hodgkin's lymphoma, B-cell 1396
Non-palpable breast disease 1017
Non-small-cell lung cancer 104, 806, 1427,
1747
NQO1 1305
NSAID drugs 1364
NSCLC 881
NTRK1 proto-oncogenes 308
Nuclear localization 1198
Nude mice 913, 931
Numbers 348
Nutritional status 1568
Observer variation 339
ODC 1041
Oesophageal cancer 429, 1557
Oesophageal squamous cell carcinomas 1892
Oesophagus 1510
Oestrogens 492, 518, 1867
Oestrogen receptors 270, 1030, 1844
Oestrone sulphatase 492
Oestrone sulphates 492
Oligoastrocytomas 1371
Oligodendrogliomas 1371
Oncogenes 315
Oncostatin M 881
Oral cancer 204, 1871
Oral leukoplakia 838
Oral SCC 838
Osteopontin 1974
Osteosarcoma 85, 1327, 1677
Ovarian cancer 436, 571, 579, 616, 621, 891,
1138, 1266, 1535, 1662
Ovarian function 1879
Ovarian tumours 760
Overall mortality 584
Over-expression 1694
Oxygenation 2009
P13 kinase 666
P14ARF
818
p16 374
P16INK4A
818
p16INK4
675
p21/WAF1 1983
1p36 823
p53 136, 171, 178, 392, 560, 571, 621, 757,
763, 913, 1145, 1709, 1892, 1939
p53 expression 270
p53 gene mutation 579, 1939
p53 gene transfer 642
p53 protein accumulation 579
p73 823
p90rsk 1041
p706Sk 1041
Paclitaxel 300, 1914
Pancreatic cancer 98, 931, 1013, 1646
Pancreatic cancer, advanced 1772
Pancreatic neoplasms 900
Pancreatic tumours 691
Papillomavirus, human 424, 1348
Papillomavirus infection, human 1332
Paramagnetism 88
Parental autoimmune diseases 1353
Parents 251
Parity 737
Pathology 760
Patient delays 742
PAX 1239
PBMC 472
PBSC 278
Peritoneum 1233
Peutz-Jeghers syndrome 1403
P-glycoprotein 1327
P-glycoprotein expression 1732
Phage displays 1808
Pharmacokinetics 767
Phase I studies 300, 767
Phase II studies 772, 1772
Phase III studies 1789
Phenacetin 1364
Phenotypes 330, 621
Phosphoglycerate mutase activity and
isoenzymes 20
Phospholipase C 16
Phosphorylation 28
Photochlor 1297
Photodynamic therapies 56, 418, 1297, 1485,
1835
Photofrin 1485
Photoimmunotherapy 56
Photosensitizers 56
Pilocytic astrocytomas 1218
Pituitary tumours 1441
Placental site trophoblastic tumours 1186
Plasminogen activator inhibitor 1 1702
Platelet-derived endothelial growth factor
1427
Platinum 436, 966
Platinum agents 34
Poly(ADP-ribose)polymerase 629
Polylysine 56
Polymerase chain reaction 157, 412, 1421,
1650
Polymorphism 535, 550, 757, 1671
Population screening 731
Population-based cohort studies 1070
Positron emission tomography 608
pRb 675
Predictive factors 560
Predisposition 871
Pregnancy 1070
Premenopausal 1920
Prevention programmes 1381, 1887
Primary tumours 1682
Prior beliefs 213
Progesterone receptors 1030
Prognosis 339, 374, 560, 1191, 1446, 1656,
1662, 1747, 1983
Prognostic factors 142, 368, 1163, 1211,
1387, 1965
Proliferating cell nuclear antigens 404
Proliferation 368, 1051, 1441, 1747
Proliferation index 167
Proliferative tumour cells 1223
Proline-directed protein kinase FA
1480
Prophylactic mastectomy 1249
Prophylactic oophorectomy 1249
Prophylaxis 1381
Prospective studies 263
Prostaglandin E2
2002
Prostate cancer 39, 112, 283, 361, 452, 858,
990, 1358, 1827, 2002
Prostate neoplasms 241
Prostate-specific antigens 361, 731
Prostate/prognostic factors 186
Prostatic cell lines 1694
Prostatic neoplasms 718
Protection, from apoptosis 981
Protein isoprenylation 905
Protein kinase 2002
Protein kinase C 34, 1063
Protein phosphorylation 142
Proteinase 931
Proteinase inhibitors 931
pS2 gene 1844
Psychotherapy 251
PTEN 1671
14q32 1801
Quality of life 263, 1789
Quantitative PCR 1682
Quantitative RT-PCR 142
Quiescent tumour cells 1223
Quinone oxidoreductase 1 1305
Racial aspects 718, 1875
Radiation 937
Radiation risks 220
Radiation-induced bystander effect 1740
2036 Keyword index
British Journal of Cancer (2000) 82(12), 2032-2036 (c) 2000 Cancer Research Campaign
Radioiododeoxyuridine 74
Radiotherapy 960, 1131, 1177, 1925
Radon 1073
Randomised clinical trials 1783
Randomised phase III trials 1138
Ras 891
Ras oncogenes 412
Ras proteins 905
Rats 74, 973, 1000
Rearrangements 308
Reasons for delaying 742
Rectal cancer 960, 1860
Ref 315
Regeneration 945
Relapse 601
Relaxation time 88
Renal cancer 550
Renal cell carcinoma 772, 812, 1063, 1851
Reoxygenation 1177
Resistance 436
Responses 1629
Restorative surgery 1131
Retinoblastoma 1875
Retinoids 452
Reverse transcription 1650
Reverse transcription polymerase chain
reaction 124, 1283
Rhabdomyosarcomas 1239
Ribosomal DNA 514
Risk factors 1332, 1358, 1593, 1795
RT-PCR 429, 571
Rural 1863
Sample sizes 213
SAPK/JNK 1827
Sarcomas 973
Scales 800
SCC Ag-1 981
SCC antigens 429
Schedules 1519
Scotland 705
Screening 381, 871, 1605, 1795
Second cancers 1344
Selection 1387
Sentinel nodes 295
Sequels 251
Serpin 981
Sexual behaviour 718
Sexual function 283, 789
Sigma-1 receptors 1965
Sigma-2 receptors 1223
Signal transduction 16
Sinusoidal perfusion failure 794
Skin SCC, human 591
Smad 1415
Smad2 1557
Smad4 1557
Smoking 204, 227, 1871
Socio-economic factors 1358
Solid tumours 1223
Soy foods 1879
SPA 881
Spain 535
Squamous cells 757
Squamous cell carcinomas 1297, 1433
SR-BP-1 1965
Stage I 1254
Stathmin 142
Stem cell factor splice variants 1453
Steroids 1312
Sterol isomerase, human 1965
STK11 1403
Stomach 1795
Streptococcus pneumoniae 1261
Stromal sarcoma 1030
Stromal-epithelial interaction 186
Structure-activity relationships 966
Substance P 480
Subtractive hybridization 1149
Sun exposure 1887
Sunbeds 1593
Sunlight 1875
Surface epithelial cells 1415
Surgery 1186
Surveillance, epidemiology and end results
(SEER) programme 1875
Survival 208, 579, 760, 782, 1254, 1339,
1387, 1568, 1747, 1863
Survival analysis 339, 1789
Sustained release 74
SV40 1677
System delays 742
Tachykinin NK1
receptor antagonists 480
Taiwan 852
Tamoxifen 1629
Tamoxifen resistance 1844
T-cells 691
Telomerase 833, 1051, 1819, 1953
Telomerase activity 601
Telomere length 601
Temozolomide 608
Testosterone 789
TGF- 865
TGF- RI 1171
TGF- RII 1171, 1557
TGF- RIII 1171
TGF-1 1171
Thalidomide 812
Thiotepa 1925
Thymidine transport 924
Thymosin-1
radioimmunoassay 584
Thyroglobulin mRNA 1650
Thyroid cancer 157, 308, 315, 1650
Time trends 1585
Tirapazamine 651, 1469
Tissue concentration 1000
Tissue factor 1974
Tissue inhibitoral metalloproteinase invasion
657
Tissue temperature 794
TNF 1000
TNF- 441, 812, 973
Tolerance 263
Tomudex 1932
Top1 913
Topoisomerase II inhibitors 1819
TP53 823
TP53 mutations 1183
Transendothelial migration 472
Translocation 330
Transplantation 81
Treatment 737, 1030
Trends 1887
Trial participation 1783
TRK oncogenes 308
Trophoblastic disease 1393
Tropical climate 1875
TS 560
Tubal ligation 1600
Tube formation 385
Tubular differentiation 1656
Tumorigenesis 9
Tumours 1474, 1513
Tumour antigens 691
Tumour blood flow 1835
Tumour cells 900
Tumour immunology 1808
Tumour markers 186, 782, 1959
Tumour metastasis 1233
Tumour models, mouse 1835
Tumour necrosis factor- 441, 812, 973
Tumour progression 65, 543, 601
Tumour proliferation markers 584
Tumour size, stage and grade 1407
Tumour stages 208, 591
Tumour suppression 459
Tumour suppressor genes 323, 459, 1123,
1671
Tumour therapy 629
Tumour volume 1004
Tumour xenografts 480
Tumour-associated proteins 981
Type 1 collagen 858
Type IV collagenases 960
UKCCSG 9002 protocols 1396
Ultrastructure 1198
Ultraviolet radiation 1344, 1593
Unique cytosolic activities 1305
uPA receptors 1702
Uranium 763
Urban 1863
Urinary tract infections 1107
Uterus 1030
Uveal melanoma 330, 818
Vaccination 1261
Vaccines 1619
Validation 800
Vascular endothelial growth factor 161, 591,
635, 1004, 1427, 1528, 1694, 1974
Vascular maturation 844
Vascular permeability 1513
Vascularization 591
Ventral tongue mucosa, rat 1764
Vindesine 488
Vinorelbine 1907
Viral involvement 1677
Vulvar cancer 295
Weight 1860
Wilms' tumour 323
X-chromosome inactivation 827
Xenografts 1844
Zoonosis 446

				

